# Evaluating Sustainability of Regional Water Resources Based on Improved Generalized Entropy Method

**DOI:** 10.3390/e20090715

**Published:** 2018-09-18

**Authors:** Ming Zhang, Jinghong Zhou, Runjuan Zhou

**Affiliations:** School of Civil Engineering, Anhui Polytechnic University, Wuhu 241000, China

**Keywords:** regional water resources, sustainability, system comprehensive evaluation, information theory, relative entropy

## Abstract

The sustainability of regional water resources has important supporting data needed for establishing policies on the sustainable development of the social economy. The purpose of this paper is to propose an assessment method to accurately reflect the sustainability of regional water resources in various areas. The method is based on the relative entropy of the information entropy theory. The steps are as follows. Firstly, the pretreatment of the evaluation sample data is required, before the relative entropy of each standard evaluation sample and evaluation grade (*SEG*) is calculated to obtain the entropy weight of each evaluation index. After this, the entropy weighted comprehensive index (*WCI*) of the standard evaluation grade sample is obtained. The function relation between *WCI* and *SEG* can be fitted by the cubic polynomial to construct the evaluation function. Using the above steps, a generalized entropy method (GEM) for the sustainable assessment of regional water resources is established and it is used to evaluate the sustainability of water resources in the Pingba and Huai River areas in China. The results show that the proposed GEM model can accurately reflect the sustainable water resources in the two regions. Compared with the other evaluation models, such as the Shepherd method, Artificial Neural Network and Fuzzy comprehensive evaluation, the GEM model has larger differences in its evaluation results, which are more reasonable. Thus, the proposed GEM model can provide scientific data support for coordinating the relationship between the sustainable development and utilization of regional water resources in order to improve the development of regional population, society and economy.

## 1. Introduction

Regional water resource sustainability (RWRS) is a state in which water resources can be recycled and renewed naturally according to the technical and economic levels and social production conditions [1,2], so that the regions can constantly provide water for industrial and agricultural production, but also protect people’s livelihood, as well as ecological and environmental factors [3,4,5]. Therefore, the assessment of RWRS is based on the comprehensive analysis of water resource characteristics, degree of guarantee, development and utilization of water resources in the basin, as well as the demand of water resources for industrial and agricultural production, people’s life and ecological environment [6,7,8]. Using the grade of RWRS, the relationship between water resources, river basin economy and population can be revealed and water resources can be reasonably and fully utilized. Thus, this allows economic construction and water resources protection to be carried out simultaneously, while social and economic sustainable development can be promoted [9,10].

When the grade of RWRS exceeds a certain threshold, it will seriously restrict regional sustainable development [11]. Therefore, it is important to accurately evaluate and diagnose regional water resources [12]. At present, the main methods for the comprehensive evaluation of the grade of RWRS include the artificial neural network method [13] (ANN), fuzzy comprehensive evaluation method [14] (FCE), Shepard method [15] (SP), etc. These methods have been used to evaluate the sustainable grade of RWRS from different perspectives. However, the evaluation results of these methods are often too close to distinguish the evaluation samples correctly and thus, are not conducive for formulating sustainable development policies [16]. In view of this problem, this paper intends to study an evaluation method based on the information entropy theory in order to increase the difference between evaluation results. This will ultimately allow us to better differentiate the sustainable evaluation results.

In information theory [17], the entropy value is usually used to measure the degree of disorder of a system, such as in a comprehensive evaluation system composed of evaluation samples and evaluation indicators [18,19,20]. The entropy method determines the weight of indicators according to the information provided by various indicators; makes full use of the difference information provided by various evaluation samples [21]; and can be used for many applications in the evaluation of water resources system or ecology system [22,23,24,25,26].

However, the entropy method only uses the different information between samples under a certain index and does not take into account the different information between the comprehensive evaluation results [27]. This may also lead to the problem of an excessively small difference degree of evaluation results, meaning that the evaluation results cannot accurately reflect the subtle differences between samples to be evaluated [28,29]. By analyzing the evaluation process of the entropy method, we found that the entropy method actually implies a hypothesis in its application. Essentially, it believes that the comprehensive evaluation value results are uniformly distributed and there is no difference between different samples, which is obviously not consistent with the target of significant differences between the comprehensive values of the actual sample. This may also be the main reason why the evaluation results of other methods are not distinguishable.

Therefore, we propose the hypothesis that when establishing the comprehensive evaluation function with the standard evaluation grade sample, it is necessary to differentiate the comprehensive evaluation results. Thus, we can use the generalized entropy weight to measure the differences in order to build the comprehensive evaluation model. In this paper, the data series of each sample under each index and the series of standard evaluation grades (*SEG*) are obtained respectively through data pretreatment [30]. After this, the relative entropy method in the information theory is used to calculate the relative entropy value (*REV*) between each index and *SEG* one by one, before the degree of proximity between each index and *SEG* is obtained successively. Relative entropy [31] (which is also called the Kullback–Leibler divergence) is a measure of how one probability distribution is different from a second reference probability distribution. In a simple case, a relative entropy value of 0 indicates that we can expect similar behavior in two different distributions.

Obviously, a closer distribution between the sample value of an index and the *SEG* indicates that the corresponding index should be given a larger weight [2,32]. According to the *REV* of each index, the entropy weight of each index is obtained by normalization in order to calculate the weighted comprehensive index (*WCI*), before the Generalized Entropy Method (GEM) is constructed.

For grading evaluation problems, the distribution of *SEG* constitutes the prior distribution of the comprehensive evaluation value [3,4]. For clustering evaluation problems, prior information of the *SEG* distribution cannot be found [2], so we could assume that *SEG* is uniformly distributed according to the principle of maximum entropy in the information theory.

In general, the purpose of this paper is to establish an assessment method to improve the discrimination degree of evaluation results. In this method, the relative entropy theory is used to measure the degree of proximity between the evaluation index series and the *SEG* series in order to calculate the weight of each index. The second part of this paper introduces the main steps of the GEM method and the two study areas of sustainable utilization of regional water resources. Section 3 shows the results of pretreatment, weight solution and evaluation, respectively. Section 4 discusses the rationality of GEM method evaluation results and the comparison with SP and other methods. This study is expected to provide guidance for coordinating the sustainability of regional water resources.

## 2. Materials and Methods

### 2.1. Generalized Entropy Method

The index system could be described as the matrix $X={\left(x_{ij}\right)}_{ni\times nj}$, where *x_ij_* is the evaluation index *j* in *i*-th region; *n_j_* is the number of evaluation indices; and *n_i_* is the total number of regions. The *SEG* could be described as $Y={\left(y_{i}\right)}_{ni}$, where *y_i_* is the standard grade of *i*-th region.

There are only a few standard evaluation grade samples in the standard grade criteria, which are not enough to establish the evaluation function. The solution of this study is to use the Monte Carlo random method to produce standard evaluation samples according to the literature [15,33]. However, the premise of using the random method to generate evaluation samples is that the evaluation index should have an upper and lower interval in each evaluation grade. Therefore, within each evaluation grade, 10 evaluation index values were randomly generated by using uniform random numbers. 

The process to establish the GEM model for the evaluation of RWRS included the following five steps.

Step 1: Data preprocessing

In this step, the data of the evaluation system will first be consistently processed, before being normalized. Firstly, all indicators are processed into consistent data of the same direction. For example, for the *j*-th indicator, the smaller the better type is; for other indicators, the bigger the better type is. Thus, the indicator *j* can be treated as the bigger the better type, which is consistent with other indicators as described in Equation (1).

(1) $$r_{ij}=\frac{\max\left(x_{ij}\right)}{x_{ij}}\left(i=1,2,\cdots ,n_{i}\right)$$

where, $r_{ij}$ represents the processed evaluation data of the *i*-th sample under the *j*-th index respectively; max(*x_ij_*) represents the maximum value of each sample in the *j*-th index. It needs to be noted that the indicators of the bigger the better type are not needed to process by Equation (1). After the consistency processing, all the indicators become the bigger the better type.

In order to calculate the relative entropy value (*REV*), it is necessary to normalize the consistent indexes series *x_ij_* and the *SEG y_i_* series. The normalization value of the *i*-th sample under *j*-th index *p_ij_* can be calculated as:

(2) $$p_{ij}=\frac{r_{ij}}{\sum_{k=1}^{n_{i}}r_{kj}}\left(i=1,2,\cdots ,n_{i};j=1,2,\cdots ,n_{j}\right)$$

The normalization value of the *i*-th sample of *SEG_i_* can be calculated as:

(3) $$SEG_{i}=\frac{y_{i}}{Sum\_y}\;\;\;(i=1,2,\cdots ,n_{i})$$

where *Sum_y* is the sum of the standard grade *y_i_*.

Step 2: Weight calculation of each indicators

First, the *REV* of each index is calculated. The approximate extent of the standard sample series *p_ij_* and series *SEG_i_* under the index *j*, *REV_j_*, can be calculated according to relative entropy [31].

(4) $$REV_{j}=\sum_{i=1}^{ni}p_{ij}\log\left(\frac{p_{ij}}{SEG_{i}}\right)\;\;\;(j=1,2,\cdots ,n_{j})$$

When the *j*-th sample series distribution *p_ij_* is the closest to that of the *SEG* series *SEG_i_*, *REV_j_* is the smallest. This indicates that the index has a greater weight in the comprehensive evaluation system and 1-*REV_j_* could be regarded as an important degree of the *j*-th index [34]. Therefore, the weight value of the *j*-th index *w_j_* can be calculated as:

(5) $$w_{j}=\frac{1-REV_{j}}{\sum_{j=1}^{nj}\left(1-REV_{j}\right)}$$

Step 3: Calculation of the weighted comprehensive index (*WCI*)

The formula for the *WCI* of the *i*-th standard evaluation sample is describes as follows:

(6) $$WCI_{i}=\sum_{j=1}^{nj}w_{j}p_{ij}$$

where *p_ij_* is the standard evaluation sample data. However, Equation (6) can also be used for the sample to be evaluated when the regional water resource sustainability evaluation is carried out.

Step 4: Determination of the evaluation function

*WCI* is a composite index that ranges from 0 to 1, which cannot yet correspond to specific sustainability levels. In order to determine the sustainability grade value of the regional water resources to be evaluated, it is also necessary to establish the mapping relationship between the series of *WCI_i_* and *SEG_i_*, which is called the regional sustainability index *SEG_i_ = f*(*WCI_i_*). By observing the scatter distribution relation between the *WCI_i_* and *SEG_i_* series, the polynomial curve or logistic curve can be used for curve fitting. In this paper, the cubic polynomial curve was used for simple calculation [16].

(7) $$SEG=a+bWCI+cWCI^{2}+dWCI^{3}$$

where *a*, *b*, *c* and *d* are the parameters of cubic polynomials, which can be solved by the optimization algorithm based on accelerated genetic algorithm [34] (AGA).

Step 5: Assessment of regional water resources sustainability

Let matrix *R* = (*r_ij_*)*_nk_*_×*nj*_ be the evaluation system of regional water resources sustainability (RWRS) assessment system, which has *n_k_* areas. According to Equations (1)–(6), the *WCI* values of each region to be evaluated are obtained. Meanwhile, the corresponding *SEG* value is calculated according to Equation (7). However, each regional *SEG* needs to be renormalized to obtain the grade of RWRS *G*_RWRS_ using Equation (8).

(8) $$G_{\mathrm{RWRS}}=SEG\times Sum\_y$$

A smaller *G*_RWRS_ of a region indicates better water resource sustainability of the region. On the contrary, a larger *G*_RWRS_ represents worse sustainable water resources and countermeasures are needed to coordinate the sustainable development of economy, society and other systems.

### 2.2. Materials

#### 2.2.1. Evaluation System

The factors that are influencing the regional water resources sustainability and China’s water supply and demand were analyzed systematically. Based on previous research [13,14,15], the evaluation index system consisting of seven evaluation indices (*X*_1_–*X*_7_) and four corresponding grade criteria were constructed (Table 1).

The indicators *X*_1_–*X*_5_ are of the type in which a higher value indicates a better result. Thus, a higher index value represents a better sustainable utilization state. The indicators *X*_6_–*X*_7_ are of the type in which a smaller value indicates a better result. Thus, a smaller value represents a better sustainable utilization state. The sustainable utilization state of grade 1 is the best, while the worst state grade corresponds to grade 4.

#### 2.2.2. Study Regions

In order to carry out the comparative analysis with other evaluation methods, we used the six regions in the Pingba district of The Hanzhong basin, as reported in study [13] and the six regions in the Huai river basin reported in studies [14,15] as examples. The proposed GEM method was used to evaluate the grade of RWRS of each region.

**Figure 1:**
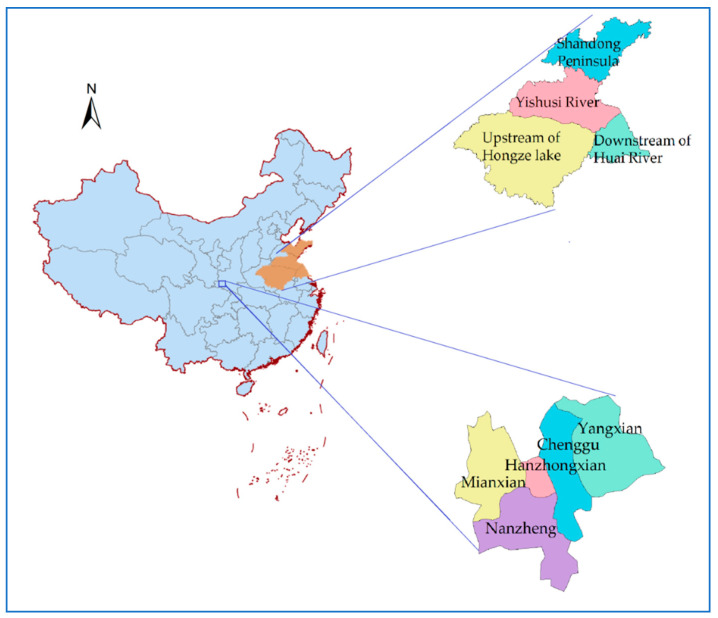
The location of the Hanzhong Basin and the Huai River regions.

The Hanzhong basin (Figure 1) is located in 106°35′–107°42′ E, 32°57′–33°15’ N. It belongs to a semi-arid and semi-humid area, which is located in the transitional zone between the subtropical zone and warm temperate zone. It has an average annual precipitation of 890 mm, an average annual runoff of 5.1 × 10^8^ m^3^ and a total groundwater resource of 7.47 × 10^8^ m^3^. Furthermore, there are many river systems and water conservancy facilities located in this basin.

The Huai River region (Figure 1) is located in north latitude 31–38° and belongs to the warm temperate semi-humid monsoon climate zone. It is composed of the two major river systems of Huai River and Yishusi River, with an average annual rainfall of 880 mm, total surface water resources of 62.1 billion m^3^ and total groundwater resources of 21.4 billion m^3^.

With the development of economy and population, the contradiction between the demand and supply of water resources in the Hanzhong basin and Huai River region has become increasingly obvious. The study on the sustainable utilization of water resources in these two regions is of great significance to further develop and utilize water resources in this region and alleviate the differences between water supply and demand.

In Table 2, Pingba covers the whole region of Mianxian, Hanzhongxian, Nanzheng, Chenggu and Yangxian. All of the Huai River basin includes upstream of Hongze Lake as well as downstream of Huai River basin and Yishusi river. The district of Huai River includes all of the Huai River basin and Shandong Peninsula.

## 3. Results

### 3.1. Random Generation of Standard Evaluation Samples and Data Preprocessing

In Grade 1, the upper limit of the water supply module *X*_4_ is set as 350 (10^4^ m^3^/km^2^) and the upper limit of water demand module *X*_5_ is set as 200 (10^4^ m^3^/km^2^). We select the upper and lower limits of the other indicators according to their physical meanings. The evaluation sample matrix *X* and the standard evaluation grade series *Y* of 40 standard evaluation samples were obtained, as shown in Table 3. Due to the confines of this paper and layout, Table 3 only provides some of the data.

In Table 3, columns 7 (*X*_6_) and 8 (*X*_7_) are the smaller and better type indicators, while Equation (1) is adopted for consistent pretreatment. For column 7 data, 2971.9 (10^4^ m^3^/person) is divided by each number. For column 8 data, we use 5.0% divided by each number. After this, all the standard evaluation samples were normalized according to Equation (2). Due to the length of calculation, the preprocessed data is not listed here.

### 3.2. Relative Entropy and Index Weight

The *REV* and the weight of each index *w_j_* are calculated, as shown in Table 4.

As shown in Table 4, the *REV* of *X*_3_ is the smallest at 0.145, which indicates that the evaluation sample distribution of this index is the closest to the *SEG* distribution. Thus, it is necessary to assign a large weight of 0.172 to *X*_3_. However, the *REV* of *X*_6_ is the largest, which indicates that the sample distribution of the index evaluation is quite different from that of the *SEG* distribution. Thus, the weight of this index is the smallest at only 0.096. After the calculation of each index weight *w_j_*, *X*_3_ plays the most important role in improving the sustainability of regional water resources, which needs to be considered in guiding policy formulation, followed by *X*_1_, *X*_2_, etc.

### 3.3. WCI and Evaluation Function

**Figure 2:**
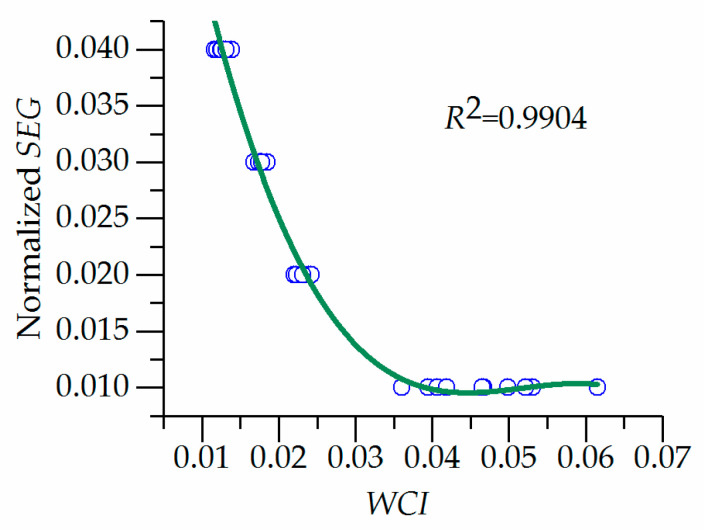
Scatter plot of weighted comprehensive index (*WCI*) and sample and evaluation grade (*SEG*).

The scatter distribution between the *WCI* and the normalized standard grade value *SEG* is observed, as shown in Figure 2.

The evaluation function is established by using cubic polynomial curve fitting and the optimization algorithm based on AGA is adopted to solve all four parameters, which is shown as follows:

(9) $$SEG=0.0827-4.3913WCI+86.565WCI^{2}-557.93WCI^{3}$$

The *SEG* of each sample was renormalized to the calculated grade using Equation (8), which was listed in Table 1, column 10, “calculated grade”. The last column of “error grade” in Table 1 is the error between the GEM calculated grade and the standard grade. From Table 1, the absolute error grade is less than 0.1 that accounts for 72.5%, which indicates a feasible evaluation function that can be used for the regional sustainable evaluation of water resources.

### 3.4. Assessment the Grade of RWRS in Two Study Regions

The index data in Table 2 of 12 sub-regions in Hanzhong and Huai River Basin were calculated by Step 1–5 successively and the evaluation grades of each region were obtained, as shown in Table 5. Table 5 also shows the results of literatures using the Artificial Neural Network method [13] (ANN), Fuzzy Comprehensive Evaluation method [14] (FCE) and Shepard similarity interpolation evaluation model [15] (SP).

Table 5 shows that the GEM method proposed in this paper is generally consistent with the results of the other three evaluation methods. Hanzhong basin is in the sustainable state of 2–3 grades and Huai river basin is between 1.5 and 2.5 grades.

## 4. Discussion

### 4.1. Discriminability Analysis

The last line in Table 5 shows the variance of the evaluation results obtained using the four evaluation methods, which can reflect the discrimination degree of the evaluation results of the four evaluation methods to a certain extent. Obviously, a greater variance represents greater discrimination. The variance results show that GEM method has the largest variance in evaluation results and is significantly statistically different to SP (*p*-value = 0.0072, which is <0.01). The variance of ANN and FCE is so small that it is difficult to distinguish the sustainable utilization status of water resources in each sub-region.

**Figure 3:**
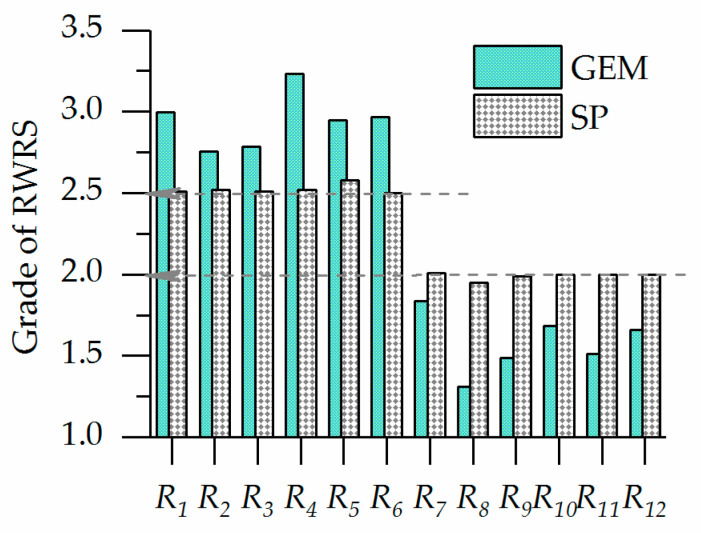
Sustainability of water resources in Hanzhong and Huai River Basin. (R_1_–R_6_ represent Hanzhong, while R_7_–R_12_ Huai River).

In order to further compare the discrimination degree of evaluation methods, the evaluation result graph of GEM and SP is drawn, as shown in Figure 3.

Figure 3 shows that GEM evaluation results are generally consistent with SP evaluation results. Both evaluation methods agree that the sustainable utilization degree of water resources in the six regions of Huai River is lower than that in the six regions of Hanzhong. This indicates that there is still a large development space in the Huai River basin, which is consistent with the literature research results [13,14,15].

However, there are still significant differences between the two evaluation models in the data distribution of the evaluation results. In six regions of Hanzhong, the GEM results ranged from 2.76 in R_2_ Hanzhongxian to 3.23 in R_4_ Chenggu. However, the SP results of these six regions are about 2.5 and the sustainable utilization of water resources varies very little from the smaller value of 2.5 in R_6_ Pingba to the largest value of 2.8 in R_5_ Yangxian, with only a difference of 0.3 in the grade.

### 4.2. Rationality Analysis of Evaluation Results

Taking the R_4_ Chenggu and R_5_ Yangxian regions of Hanzhong basin as examples, the rationality of the GEM and SP evaluation results is analyzed.

Firstly, the rationality of the evaluation results is judged according to the values of each single index in R_4_ Chenggu. In Table 2, the index of irrigation rate in R_4_ Chengu is 31.3%, which is greater than grade 3; *X*_2_ is 25.8%, which is greater than grade 3; *X*_3_ is 48.4%, which is less than but close to grade 3; *X*_4_ is 36.7 × 10^4^ m^3^/km^2^, which is larger than grade 3 and close to grade 4; *X*_5_ is 76.5 × 10^4^ m^3^/km^2^, which is less than grade 3 but greater than grade 2; *X*_6_ is 1102.6 × 10^4^ m^3^/person, which is less than grade 2 and greater than grade 1; *X*_7_ is 2%, which is grade 1.Three of the seven indicators are greater than grade 3, two are close to grade 3 and two are close to grade 1. Therefore, it is reasonable to use the GEM model to evaluate the sustainable utilization grade of water resources in R_4_ Chenggu as a grade of 3.23. However, the evaluation grade of SP model is 2.52, which is obviously not as reliable as the evaluation results from GEM in terms of the characteristic values of a single index in R_4_ Chenggu.

Secondly, the rationality of the GEM and SP methods is judged by comparing the evaluation results of the two regions. In the SP model, the RWRS grade in R_4_ Chenggu and R_5_ Yangxian regions is evaluated as 2.52 and 2.58, respectively, while the GEM model is evaluated as 3.23 and 2.95, respectively. GEM and SP have opposite results of the grade of RWRS in these two areas. Further analysis is needed to determine which method is more reasonable.

The characteristic values of each indicator in Table 2 are compared. For the bigger the better type indicators *X*_1_–*X*_5_, the index characteristic values of R_4_ Chenggu are smaller than the corresponding index characteristic values of R_5_ Yangxian, which indicates that the sustainable utilization of water resources in R_4_ Chenggu is lower than that of R_5_ Yangxian. For the smaller the better type indicator *X*_6_, the value of R_5_ Yangxian is 1032.7 × 10^4^ m^3^/person, which is less than 1102.6 × 10^4^ m^3^/person in R_4_ Chenggu. This also indicates that the grade of RWRS of R_4_ Chenggu is lower than that of R_5_ Yangxian. On index 7, the values of the two regions are the same. Through the comparative analysis of two regional indicators, we suggest that the grade of RWRS in R_4_ Chenggu is lower than that in R_5_ Yangxian. Thus, the reasonable evaluation result should be that *G*_RWRS_ of R_5_ Chenggu > *G*_RWRS_ of R_6_ Yangxian. Obviously, the evaluation result from GEM is more reasonable than that from SP. The performance of GEM and SP in Huai River Basin is similar to that of Hanzhong Basin. Compared with the SP model, the GEM model has better differentiation results and there is no logical irrationality.

## 5. Conclusions

In order to improve the rationality and discriminability of the evaluation results related to the sustainable utilization of regional water resources, we used the relative entropy method in the information theory to establish a generalized entropy method (GEM) consisting of five steps and obtained the following conclusions.

(1) The proposed GEM method can significantly improve the differentiation of the evaluation results. Compared with the evaluation results of SP and other evaluation methods, the variance of the GEM method evaluation results is significantly higher than that of other methods. The evaluation results are more distinguished.

(2) The GEM method can correctly reflect the sustainable state of regional water resources and the evaluation result is more reasonable. GEM evaluates the six regions of Hanzhong Basin to have a grade around 3.0, while the evaluation results of Huai River Basin are all less than 2. The water resource sustainability of Huai River Basin is lower than that of Hanzhong Basin, which show a more sustainable state of water resources than Hanzhong Basin.

(3) GEM is more reasonable than SP in evaluating the sustainability of water resources. The analysis from two aspects, including the rationality of the single regional grade and two regional grade rankings, shows that the evaluation results from GEM are more accurate and there is no sorting error.

In the proposed GEM evaluation method, the index weight calculated by the difference of evaluation samples is called the objective weight. However, this objective weight has not considered the subjective weight determined by experts according to the meaning of each index. In future studies, the subjective weight method, such as the analytic hierarchy process, can be considered to integrate the objective weight to obtain more reliable evaluation results.

## Figures and Tables

**Table 1:** Index system and corresponding grade criteria for evaluating regional water resource sustainability (RWRS) in China.

Evaluation Index	Water Resources Sustainability Grade Criterion			
	Grade 1	Grade 2	Grade 3	Grade 4
*X*_1_ rate of irrigation (%)	≥60	45	35	≤20
*X*_2_ rate of water resources utilization (%)	≥60	45	35	≤20
*X*_3_ rate of water resources development (%)	≥70	55	45	≤30
*X*_4_ modulus of water demand (10^4^m^3^/(km^2^))	≥100	80	60	≤40
*X*_5_ modulus of water supply (10^4^m^3^/(km^2^))	≥100	80	60	≤40
*X*_6_ water supply per capita(10^4^m^3^/person)	≤1000	1750	2250	≥3000
*X*_7_ rate of ecological water consumption (%)	≤2	3	4	≥5

**Table 2:** Characteristics of water resources sustainable status in the Hanzhong and Huai River regions.

Regions	Sub regions	*X*_1_	*X*_2_	*X*_3_	*X*_4_	*X*_5_	*X*_6_	*X*_7_
Hanzhong Basin [13]	R_1_ Mianxian	39.1	22.5	43.5	95.5	46.0	1006.6	2
	R_2_ Hanzhongxian	37.6	26.7	50.3	98.4	50.7	885.2	2
	R_3_ Nanzheng	40.3	25.6	49.5	106.8	53.9	1225.8	2
	R_4_ Chenggu	31.3	25.8	48.4	76.5	36.7	1102.6	2
	R_5_ Yangxian	32.7	28.9	53.0	95.2	37.7	1032.7	2
	R_6_ Pingba	35.8	25.7	48.7	92.7	44.6	1041.4	2
Huai River [14,15]	R_7_ Upstream of Hongze Lake	55.3	51.1	42.9	13.5	12.9	244.1	1
	R_8_ Downstream of Huai River	90.5	71.5	94.2	29.2	43.3	495.8	1
	R_9_ Yishusi River	69.1	72.1	68.4	20.0	26.7	319.3	1
	R_10_ All of Huai River basin	63.4	59.3	55.8	17.2	23.7	296.8	1
	R_11_ Shandong Peninsula	67.2	59.3	53.7	12.4	15.4	222.6	1
	R_12_ District of Huai River	64.1	59.3	55.5	16.3	22.2	283.8	1

**Table 3:** Results of standard evaluation samples and generalized entropy method (GEM) assessment.

No.	*X*_1_	*X*_2_	*X*_3_	*X*_4_	*X*_5_	*X*_6_	*X*_7_	Standard Grade	Calculated Grade	Error Grade
1	2	3	4	5	6	7	8	9	10	11
1	86.3	86.1	81.2	109.4	155.9	565.8	0.3	1	0.96	0.041
2	89.3	64.2	81.3	308.8	143.3	978.4	1.4	1	0.99	0.008
3	65.6	79.3	89.4	240.7	128.8	726.5	0.2	1	1.01	–0.007
4	85.0	99.8	85.2	285.4	196.0	483.1	1.3	1	0.96	0.042
5	84.3	66.4	84.1	238.7	114.2	318.3	0.3	1	0.98	0.021
6	60.4	72.6	70.9	177.0	137.6	596.7	0.8	1	1.07	–0.074
7	64.0	79.9	98.6	346.4	126.5	720.2	1.7	1	0.98	0.024
8	96.7	60.9	96.6	168.0	177.7	88.7	0.7	1	1.03	–0.026
9	86.8	86.3	90.6	330.4	102.0	154.9	1.1	1	1.00	0.001
10	99.2	91.6	80.8	310.3	135.6	947.4	1.7	1	0.96	0.036
…	…	…	…	…	…	…	…	…	…	…
30	39.5	44.6	46.5	60.2	70.6	1765.5	3.3	3	2.88	0.122
31	32.2	24.6	35.5	43.2	53.6	2338.8	4.4	4	3.94	0.062
32	31.6	32.3	42.2	44.2	55.1	2971.9	4.7	4	3.71	0.290
33	22.3	22.9	34.8	54.3	44.6	2765.9	4.6	4	4.26	–0.257
34	21.7	33.8	34.9	41.3	55.3	2386.9	4.7	4	3.98	0.019
35	25.8	20.3	37.4	58.4	55.9	2786.8	4.8	4	4.03	–0.034
36	28.4	20.3	33.0	52.4	54.5	2454.9	4.3	4	4.08	–0.077
37	33.1	20.0	34.1	48.1	41.0	2615.4	4.2	4	4.17	–0.167
38	24.7	21.7	41.7	57.9	47.6	2651.4	5.0	4	4.05	–0.048
39	26.8	26.8	34.2	52.8	52.7	2911.0	4.8	4	4.02	–0.023
40	33.5	29.8	37.5	42.6	46.7	2428.6	4.7	4	3.87	0.126

**Table 4:** Results of relative entropy value (*REV*) and weight index.

	*X*_1_	*X*_2_	*X*_3_	*X*_4_	*X*_5_	*X*_6_	*X*_7_
*REV*	0.179	0.182	0.145	0.309	0.175	0.522	0.511
1-*h_j_*	0.821	0.818	0.855	0.691	0.825	0.478	0.489
*w_j_*	0.165	0.164	0.172	0.139	0.166	0.096	0.098

**Table 5:** Results of the grade of RWRS by GEM method.

Sub Regions	ANN [13]	FCE [14]	SP [15]	This Work
R_1_ Mianxian	2.56	-	2.51	3.00
R_2_ Hanzhongxian	2.69	-	2.52	2.76
R_3_ Nanzheng	2.73	-	2.51	2.78
R_4_ Chenggu	2.37	-	2.52	3.23
R_5_ Yangxian	2.57	-	2.58	2.95
R_6_ Pingba	2.56	-	2.50	2.97
R_7_ Upstream of Hongze Lake	-	2.29	2.01	1.84
R_8_ Downstream of Huai River	-	2.10	1.95	1.31
R_9_ YishusiRiver	-	2.36	1.99	1.49
R_10_ All of Huai River basin	-	2.29	2.00	1.68
R_11_ Shandong Peninsula	-	2.49	2.00	1.51
R_12_ District of Huai River	-	2.35	2.00	1.66
Variance	0.126	0.128	0.279	0.734
